# AI-Driven Drug Target Screening Platform Identified Oncogene CACNA2D1 Activated by Enhancer Infestation in Epstein-Barr Virus-Associated Nasopharyngeal Carcinoma

**DOI:** 10.3390/ijms26104697

**Published:** 2025-05-14

**Authors:** Dittman Lai-Shun Chung, Geoffrey Ho Duen Leung, Songran Liu, Sarah Wing Yan Lok, Ying Xin, Yunfei Xia, Alex Zhavoronkov, Frank W. Pun, Wai-Tong Ng, Wei Dai

**Affiliations:** 1 Department of Clinical Oncology, University of Hong Kong, Hong Kong SAR, China; u3007476@connect.hku.hk (D.L.-S.C.); liusr@hku.hk (S.L.); ngwt1@hku.hk (W.-T.N.); 2 Insilico Medicine Hong Kong Ltd., Unit 310, 3/F, Building 8W, Hong Kong Science and Technology Park, Hong Kong SAR, China; geoffrey.leung@insilico.com (G.H.D.L.); sarah.lok@insilico.com (S.W.Y.L.); jessica.xin@insilico.com (Y.X.); alex@insilico.com (A.Z.); 3 Department of Radiation Oncology, Sun Yat-Sen University Cancer Centre, Guangzhou 510060, China; xiayf@sysucc.org.cn; 4 State Key Laboratory of Oncology in South China, Collaborative Innovation Center for Cancer Medicine, Guangdong Key Laboratory of Nasopharyngeal Carcinoma Diagnosis and Therapy, Sun Yat-Sen University Cancer Center, Guangzhou 510060, China; 5 Insilico Medicine US Inc., 1000 Massachusetts Avenue, Suite 126, Cambridge, MA 02138, USA

**Keywords:** artificial intelligence (AI) platform, Epstein-Barr virus (EBV), nasopharyngeal carcinoma (NPC), drug target screening

## Abstract

The management of nasopharyngeal cancer (NPC) is rapidly evolving, with immune checkpoint inhibitors emerging as a prominent treatment approach. However, drug development targeting specific molecular and cellular abnormalities in NPC has slowed. Recent advancements in artificial intelligence (AI) and bioinformatics, particularly those integrating multi-omics data, offer a more effective alternative to traditional in vitro screening methods for identifying clinically actionable targets in NPC. Through a combination of multi-omics analyses and AI-driven screening, we identified CACNA2D1 as a novel cancer-cell-specific therapeutic target in NPC. Our research indicates that exploiting Epstein–Barr virus (EBV) tethering increases H3K27 acetylation near the *CACNA2D1* promoter. Analysis of clinical specimens revealed significant upregulation of CACNA2D1 at both the transcriptional and translational levels (*p*-value < 0.01). Functional studies demonstrated that the mouse tumour size shrank by one-third upon the depletion of CACNA2D1, and there was an 85% reduction in cancer cell growth through the blockage of enhancers, while the presence of CACNA2D1 conferred a survival advantage during NPC tumour development. These findings highlight the potential of CACNA2D1 as a promising target for therapeutic intervention in NPC.

## 1. Introduction

Nasopharyngeal carcinoma (NPC) is a malignancy originating from the epithelial cells of the nasopharynx and has a particularly high prevalence in Southeast Asia and southern China [1,2]. NPC is classified into several subtypes based on histological characteristics, including keratinising, basaloid, and non-keratinising [3]. The keratinising subtype is less common and is characterised by the presence of keratin production in tumour cells [4]. Basaloid squamous cell carcinoma, another subtype, shows distinct histological features and has different prognostic implications [5]. The non-keratinising subtype can be further divided into undifferentiated and differentiated subtypes, and the former is closely associated with Epstein–Barr virus (EBV) infection [6].

The clinical significance of NPC is underscored by its epidemiology and association with EBV. EBV is closely linked to NPC pathogenesis, where it is seldom detected in normal nasopharyngeal epithelial tissues [7]. EBV infection is considered an early event in NPC pathogenesis, stimulating neoplastic transformation through various molecular mechanisms, including the inactivation of tumour suppressor genes and the activation of oncogenes [8,9,10].

Genetic and epigenetic alterations are pivotal in NPC development. For instance, polymorphic deletions in detoxification genes and single-nucleotide polymorphisms in the ITGA9 gene at 3p21.3 have been associated with NPC [11,12]. Epigenetic modifications, such as DNA methylation and histone modifications, also play a significant role. For example, tumour suppressor genes, particularly those located on chromosome 3p21, are frequently methylated in NPC [13]. Enhancer infestation, a phenomenon where enhancer elements are hijacked to activate oncogenes, also plays a critical role in NPC. Enhancer elements are regulatory DNA sequences that can significantly influence gene expression. In cancer, these elements can be co-opted to drive the expression of oncogenes, contributing to tumorigenesis [14,15]. Previous studies have highlighted the role of enhancer infestation in various cancers, including NPC, where it can lead to the aberrant activation of the genes involved in cell proliferation [16,17].

**Figure 1:**
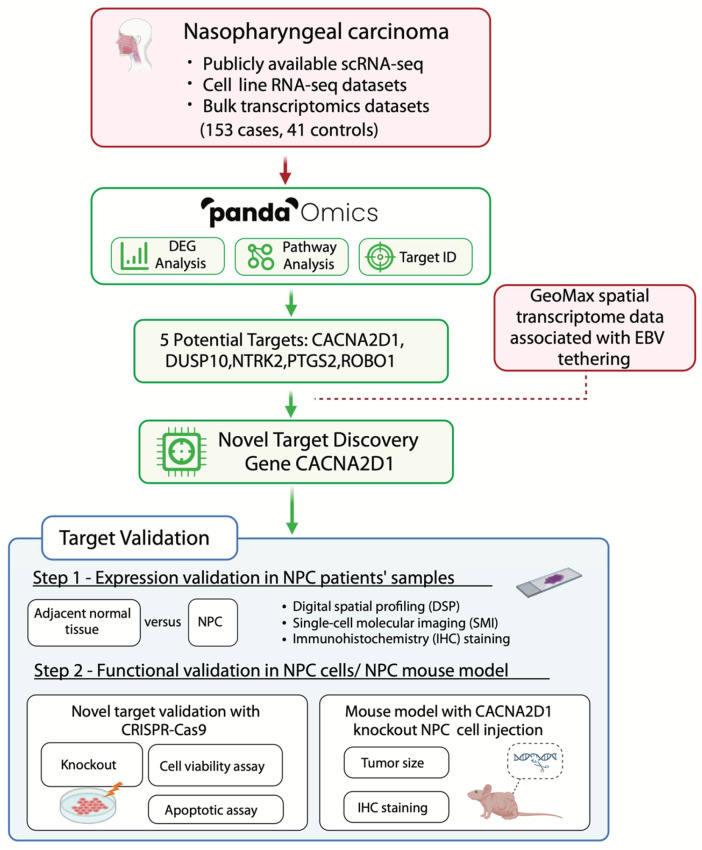
Study workflow for artificial intelligence (AI)-driven therapeutic targets identification for NPC. Case–control comparisons were performed to analyse the transcriptomic changes between NPC and control samples in each of the four selected bulk RNA-seq datasets. Subsequently, the differentially expressed genes of the Epstein–Barr virus-positive (EBV+) NPC cell lines and NPC cell lines, in comparison with normal cell lines, were analysed using RNA-seq data. The intersection of differentially expressed genes from bulk transcriptomics datasets and cell line RNA-seq datasets was input into PandaOmics, which is a generative AI platform for further target prioritisation. Following scoring and filtering, five targets—PTGS2, CACNA2D1, DUSP10, NTRK2, and ROBO1—were shortlisted. GeoMax spatial transcriptome data associated with EBV-tethering-regulated epigenome further narrowed down the five potential targets to the most promising one, CACNA2D1. This target was validated by the elevated expression in NPC FFPE patients’ samples and through in vitro and in vivo functional assays.

In the context of NPC, pinpointing specific therapeutic targets poses a significant challenge due to the intricate interplay of genetic, epigenetic, and viral factors [18]. Our study aims to tackle this challenge by employing artificial intelligence (AI)-driven drug target screening methodologies. The integration of AI into drug discovery has been transformative, enabling the rapid and precise identification of therapeutic targets [19]. AI-driven platforms surpass traditional methods by facilitating the analysis of vast datasets, identifying novel targets with greater accuracy, and potentially reducing both the time and costs associated with drug development [20]. In this study, we leveraged PandaOmics alongside comprehensive transcriptomics, epigenomics, and EBV–host genome interactions to identify key therapeutic targets in NPC (Figure 1). The platform supports therapeutic target discovery through an intuitive interface, utilising 23 disease-specific models and incorporating biological knowledge graphs and large language models for precise analysis [21]. PandaOmics has demonstrated its efficacy in identifying critical therapeutic targets, thereby expediting the development of effective treatments for various diseases [22,23,24,25].

## 2. Results

### 2.1. AI-Driven Platform Identifies Novel Therapeutic Targets

A total of nine publicly available bulk RNA sequencing (RNA-seq) transcriptomic datasets were first identified on the GEO database (Supplementary Table S1). Since the samples obtained from the nasopharynx are commonly prone to low tumour purity due to immune cell invasion [26,27], dataset selection criteria were based on tumour purity, which was achieved at an acceptable level to ensure that the downstream analyses would be less biased by infiltrating immune cells. An algorithm called Estimation of STromal and Immune cells in MAlignant Tumours using Expression data (ESTIMATE) was utilised to estimate the tumour purity in each of the nine datasets based on their expression signature to infer the fraction of stromal and immune cells in the tumour [28]. Finally, four datasets (GSE34573 [29], Tay et al. [30], GSE118719 [31] and GSE12452 [32]) with an estimated median tumour purity of 0.6 or above were selected for further analyses (Supplementary Figure S1A, Supplementary Table S1).

Next, case–control comparisons were performed using linear regression to compare the transcriptomic changes between NPC and control samples in each of the four selected bulk RNA sequencing (RNA-seq) datasets. Meta-analysing addressing the effect sizes (logFC) of the four datasets showed that a total of 1431 genes were consistently upregulated in all four datasets, whereas 1040 genes were consistently downregulated (Supplementary Tables S2 and S3). Gene Set Enrichment Analysis (GSEA) was performed for each of the four bulk RNA-seq datasets on the 50 human hallmark gene sets obtained from the Human Molecular Signatures Database [33]. While it is well known that the NF-kB signalling pathway is constitutively upregulated during NPC development [34,35], our hallmark meta-analysis of the results further showed that the E2F targets, epithelial–mesenchymal transition, and multiple processes involved in immune responses were activated. In contrast, oestrogen responses and xenobiotic metabolism were shown to be inhibited in NPC samples (Supplementary Figure S1B).

**Figure 2:**
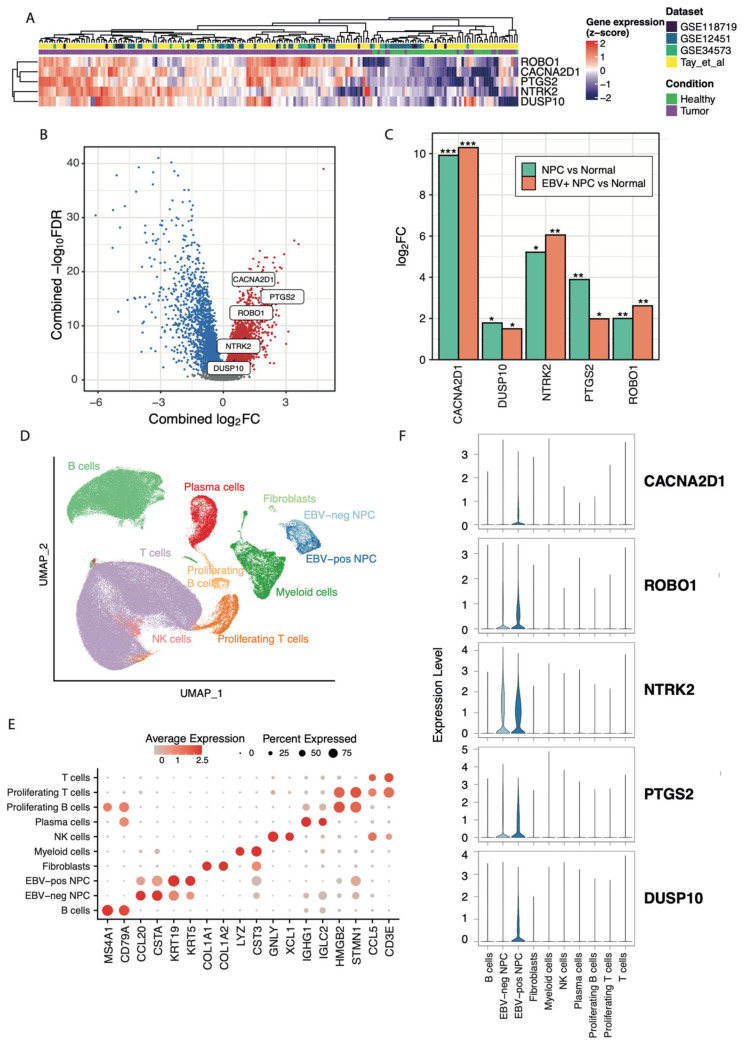
Bulk and scRNA-seq data for shortlisted target genes. (**A**) The expression profiles of the five shortlisted targets across the NPC (n = 153) and healthy samples (n = 41) in four bulk RNA-seq datasets (GSE34573 [29], Tay et al. [30], GSE118719 [31], and GSE12452 [32]). (**B**) Volcano plot representing the combined log2FC values and an FDR-adjusted *p*-value < 0.05 in NPC vs. control comparisons for all genes measured in the four bulk RNA-seq datasets. The five shortlisted target genes are highlighted. To capture all statistically significant genes from both microarray and RNA-seq data, genes with an FDR-adjusted *p*-value < 0.05 were considered significantly differentially expressed instead of using a fixed log2FC threshold [42]. Red: significantly upregulated genes; blue: significantly downregulated genes; and grey: non-significantly dysregulated genes. (**C**) The log2FC values of the shortlisted target genes in RNA-seq data using NPC cell lines. EBV+ NPC cell lines showed a total of 3146 significantly differentially expressed genes, with 1732 upregulated and 1414 downregulated genes. Secondly, the NPC cell lines showed a total of 3,092 significantly differentially expressed genes; among them, 1898 were upregulated and 1194 were downregulated. The colours of the bar plot represent either EBV+ NPC or all NPC samples versus controls. * *p*-value < 0.05; ** *p*-value; < 0.01; *** *p*-value < 0.001. (**D**) The UMAP visualisation of cells annotated by cell types in the scRNA-seq data obtained from the NPC samples available from three publicly available datasets after batch effect correction and filtering. (**E**) The top two markers are used to annotate each cell type in the scRNA-seq data. (**F**) The normalised expression levels of each of the five shortlisted target genes in the 10 annotated cell types.

We tried to prioritise the study genes via NPC cell line bulk RNA-seq data. Conditional comparisons for differentially expressed genes in EBV+ NPC versus EBV- NPC and EBV+ NPC versus normal control were conducted, showing that 1280 and 778 genes were commonly upregulated and downregulated, respectively. By overlapping differentially expressed genes in the NPC cell lines with genes identified from the four bulk RNA-seq datasets mentioned above, a total of 176 upregulated and 102 downregulated genes were revealed (Supplementary Tables S4 and S5). Meta-analysis was then performed for target prioritisation on PandaOmics. The process of target prioritisation using PandaOmics is described in Materials and Methods. After scoring and filtering, the top 100 novel targets were shortlisted for further selection. These targets were selected based on (i) their expression profiles across the four bulk RNA-seq datasets, (ii) their expression profiles in NPC cell lines, and (iii) literature reviews on safety and mechanisms of action (Supplementary Table S6). Consequently, a proof-of-concept (POC) target (PTGS2) and four novel targets (CACNA2D1, DUSP10, NTRK2, and ROBO1) were shortlisted (Table 1). These targets were significantly upregulated across all case–control comparisons either in the publicly available transcriptomics datasets or the cell line experiments (Figure 2A–C).

**Table 1:** Prioritisation and selection of five targets.

Gene	NPC vs. Control				Proposed Therapy	Protein Family	Tissue Specificity (HPA)	Biological Processes	# Clinical Trials	Any Clinical Trials for Cancers	Gene–Disease Association
	Rank	Median LFC	%	%							
			Up	*p* < 0.05							
Investigational target for NPC											
*PTGS2*	5	2.47	100	75	Antagonist	Oxidoreductase	Enhanced in the bone marrow, seminal vesicle, and urinary bladder	Biosynthesis and metabolism of fatty acid, lipid, and prostaglandin	5104	Yes	Involved in three clinical trials (max. P3) for NPC (not a novel target for NPC).
Potential novel targets for NPC											
*NTRK2*	46	1.61	100	100	Antagonist	Receptor kinase	Enhanced in the brain and thyroid gland	Differentiation and neurogenesis	211	Yes	LMP1 promotes NPC metastasis through NTRK2-mediated anoikis resistance. NTRK2 downregulation inhibits the metastasis of LMP1-positive NPC [36].
*CACNA2D1*	10	2.5	100	100	Antagonist	Ion channel	Enhanced in the skeletal muscle and tongue	Calcium transport, ion transport, and transport	922	None *	Enhanced radioresistance in cancer stem-like cells [37]; critical for HCC stemness and predictive of poor prognosis for HCC [38,39].
*ROBO1*	38	2.13	100	100	Antagonist	Immunoglobulin	Low tissue specificity	Chemotaxi, differentiation, and neurogenesis	0	None	Reported to promote anti-tumor activities upon ROBO1 knockout/knockdown.
*DUSP10*	171	0.82	100	75	Antagonist	Esterase	Enhanced in the liver	N/A	0	None	DUSP10 inhibition in esophagus cancer cells increases apoptosis after irradiation [40]. DUSP10 is a regulator of YAP1 activity, promoting cell proliferation and colorectal cancer progression [41].

* There were clinical trials related to CACNA2D1-associated drugs i.e. gabapentin and pregabalin but they were not studying the anti-tumor effects of drugs on cancer patients but the effects on pain reduction; N/A: No retrievable biological processes were found for DUSP10 due to its novelty.

Identifying five shortlisted targets by the AI platform, we aim to validate these targets at the single-cell transcriptome level. A total of three single-cell RNA-seq (scRNA-seq) datasets were retrieved from published studies, which were those obtained from Bei et al. [43], Guan et al. [44], and Ma et al. [45]. Extracting the cases from these studies and combining the datasets with correction for batch effects, a total of 10 cell clusters were identified and annotated using the top differentially expressed surface markers, including B cells, NPC without detectable EBV (EBV- NPC) cells, EBV+ NPC cells, fibroblasts, myeloid cells, natural killer (NK) cells, plasma cells, proliferating B cells, proliferating T cells, and T cells (Figure 2D,E) (Supplementary Figure S1C). We profiled the expression level of the five shortlisted targets in each cell type, showing that *NTRK2, PTGS2*, and *ROBO1* were expressed in both EBV+ and EBV- NPC cells, whereas *CACNA2D1* and *DUSP10* were specifically expressed in EBV+ NPC cells. These five targets were all specifically expressed in NPC cells from NPC patients, suggesting that the therapeutic inhibition of these targets could potentially minimise the side effects due to their specific expression in NPC cells (Figure 2F).

### 2.2. Enhancer Infestation at CACNA2D1 and ROBO1

**Figure 3:**
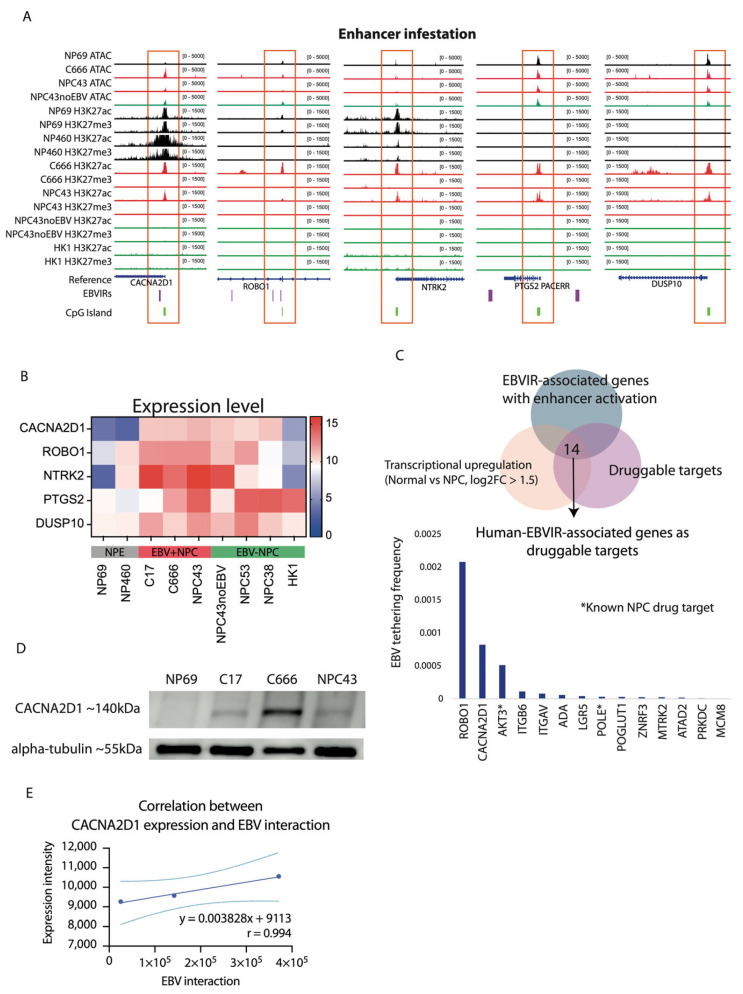
The EBV interactions and enhancer infestation of five shortlisted genes. (**A**) Chromatin accessibility, histone modifications, and the identification of EBVIRs for CACNA2D1, ROBO1, NTRK2, PTGS2, and DUSP10 visualised in the IGV browser. The ATAC-seq profiles’ chromatin accessibility status and the CUT&RUN-seq profiles’ enhancer (H3K27ac) and suppressor (H3K27me3) histone marks are highlighted for NPE (black), EBV+ NPC (red), EBV- NPC (green). (**B**) Gene expression levels for CACNA2D1, ROBO1, NTRK2, PTGS2, and DUSP10. The bulk RNA-seq with normalised read counts was evaluated for NPE and NPC cell lines. Red indicates higher expression levels and blue indicates lower expression levels in the heatmap. (**C**) Identification of human-EBVIR-associated genes as druggable targets. The Venn diagram identified 14 upregulated genes from GeoMax spatial transcriptome data associated with EBV tethering that can be potentially targeted by drugs. The bar plot lists the EBV-tethering frequency of these 14 druggable target genes. * indicates that genes are targetable by NPC drugs. (**D**) The protein expression levels for CACNA2D1. Western blot was carried out to evaluate the protein expression of CACNA2D1 at ~140 kDa in one normal NPE and three EBV+ NPC cell lines, with an equal amount of alpha-tubulin, 55 kDa, as housekeeping. (**E**) The correlation between protein expression level and EBV-tethering events. We captured the total number of EBV cross-interaction events with the human genome from modified Hi-C data. We used ImageJ (version: Java 8, 1 December 2023) to quantify the intensity of CACNA2D1 protein expression in C17, C666, and NPC43 from the Western blot result. Pearson correlation indicates a strong positive relationship between CACNA2D1 protein expression and EBV-tethering events: r = 0.994, y = 0.0038x + 9113. The dashed line indicates the 95% confidence interval (CI).

Our previous study has demonstrated that NPC has a lower mutational burden than other cancer types [46]. Whole-exome sequencing (WES) revealed a single somatic mutation (A1945G) on the *CACNA2D1* and no mutation on *ROBO1*, *NTRK2*, *PTGS2*, and *DUSP10* [47]. It detected 27.8% (60/216) for CACNA2D1, 16.2% (35/216) for ROBO1, and 28.2% (61/216) cases for NTRK2 copy number variation among shortlisted targets [48]. In particular, the detected copy number variation in *CACNA2D1* showed no significant difference in its transcription expression (Supplementary Figure S2A). Ruling out aberrant gene regulation caused by genomic alteration, we further examined the epigenomic alteration, investigating the chromatin accessibilities and histone modifications using ATAC-seq and CUNT&RUN-seq. Open chromatin accessibility was observed at *CACNA2D1*, *ROBO1*, *NTRK2*, *PTGS2*, and *DUSP10*. Additionally, H3K27 acetylation (H3K27ac) was found to be located at the promoter region of these five candidates. Furthermore, we identified that EBV-interacting regions (EBVIRs) using modified Hi-C data, capturing the EBV episomes cross-interacting with the human DNA genome, were associated with enhancer infestation in *CACNA2D1* and *ROBO1* (Figure 3A). It suggests that EBV episomes could potentially hijack the enhancer regions in the human genome to enhance their transcriptional activity. To evaluate their transcriptional activity, we used the RNA-seq data from cell lines to assess the expression level of these EBVIR-associated genes. The expression of five candidates showed transcriptional upregulation. In particular, the upregulation of *CACNA2D1* was found in six out of seven NPC cell lines, and two normal control epithelial cell lines had less transcriptional activity (Figure 3B). We overlapped EBVIR-associated genes with enhancer infestation, which were transcriptionally upregulated in NPC (PanCK-enriched segment) compared to normal regions (log2 fold change (log2FC) ≥ 1.5 and *p*adj ≤ 0.05), using GeoMax spatial transcriptome data and an AI-driven platform. There are 14 genes that are potential NPC druggable targets; *ROBO1* and *CACNA2D1* were ranked as the top two candidates with which EBV episomes are likely to tether (Figure 3C). Targeting the aberrant activation of PI3K/Akt signal transduction, AKT3 has previously been reported as a known NPC drug target [49]. ROBO1 has previously been shown to be related to cancer cell proliferation, migration, apoptosis, and angiogenesis [50]. CACNA2D1 is involved in voltage-gated calcium channel activity and regulates tumour growth via the apoptosis signalling pathway in gastric cancer [51]. We also examined the protein expression level in these five candidates. Three EBV+ NPC cell lines showed protein expression of CACNA2D1, but expression was absent in normal control NP69 cells, indicating that CACNA2D1 could be a good therapeutic target (Figure 3D). On the other hand, ROBO1 was upregulated in both normal control and NPC cells and may not be a good target candidate (Supplementary Figure S2B). This CACNA2D1 protein expression level is positively correlated with the EBV-tethering events (r = 0.994) (Figure 3E). This suggests that the increased expression of CACNA2D1 could be regulated by EBV episomes, tethering to the promoter region of CACNA2D1.

### 2.3. Aberrant Upregulation of CACNA2D1 in NPC

**Figure 4:**
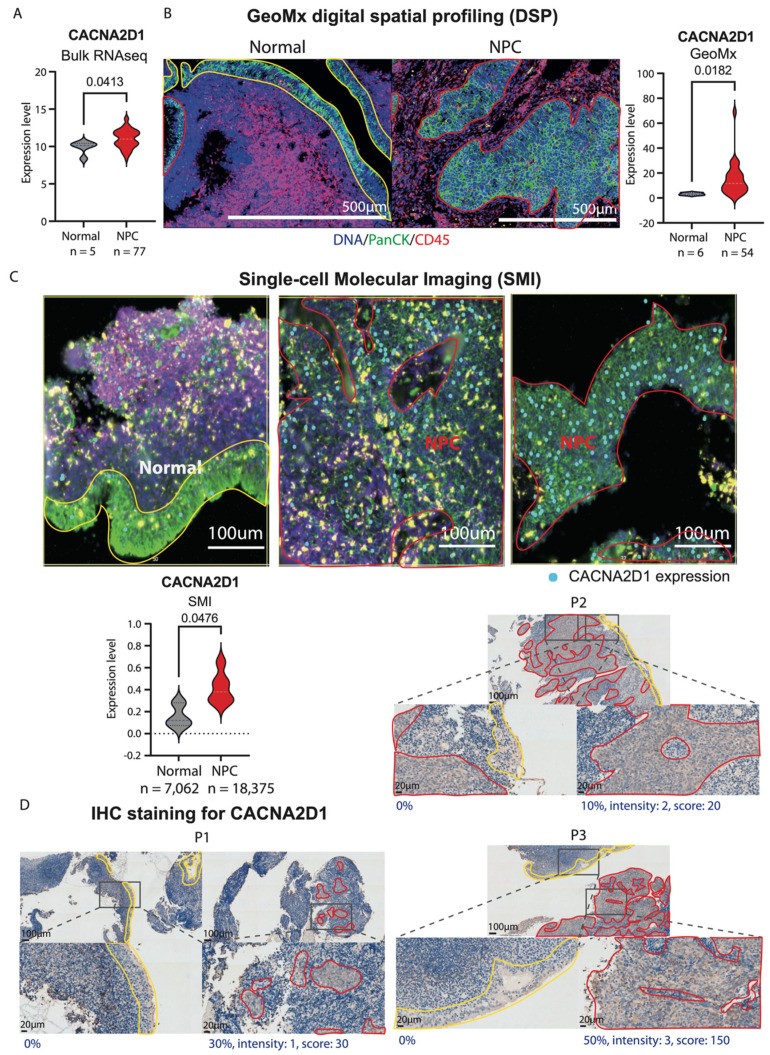
Upregulation of CACNA2D1 in digital spatial profiling (DSP), single-cell molecular imaging (SMI), and immunohistochemistry (IHC) staining in clinical specimens. (**A**) Evaluation of CACNA2D1 expression level by bulk RNA-seq from the Guangzhou cohort. With normal (n = 5) and NPC tumour (n = 77) clinical samples from GSE102349, the transcriptional level of *CACNA2D1* was evaluated. An unpaired *t*-test was performed to evaluate the statistical significance. (**B**) Quantification of CACNA2D1 protein expression levels via GeoMx digital spatial profiling (DSP). Normal (n = 6) and NPC specimens (n = 54) were stained with DAPI (blue), PanCK (green), and CD45 (red). The expression level was quantified at PanCK+ segments. The red frames indicate tumour segments, and the yellow frames indicate normal adjacent segments evaluated by a pathologist. An unpaired t-test was performed to evaluate the statistical significance. (**C**) Quantification of the *CACNA2D1* expression level via single-cell molecular imaging (SMI). Two NPC and one normal clinical sample were analysed with SMI. The cyan colour dots indicate the detection of *CACNA2D1* expression. An unpaired t-test was performed to evaluate the statistical significance. (**D**) Validation of CACNA2D1 by immunohistochemistry (IHC) staining. Three clinical FFPE patient slides were stained with the CACNA2D1 (MA3-921) antibody. The red frames indicate tumour segments, and the yellow frames indicate normal adjacent segments. The IHC scoring was evaluated by a pathologist.

As described above, CACNA2D1 was shown to be consistently upregulated in the four selected publicly available bulk RNA-seq cohorts, three scRNA-seq datasets, and the previously established NPC cell lines. Further to this, we clinically and independently validated the expression level of CACNA2D1 from bulk RNA-seq, where patient biopsies were collected from four public hospitals (Queen Mary, Queen Elizabeth, Princess Margaret, and Pamela Youde Nethersole Eastern Hospitals) in Hong Kong. CACNA2D1 was significantly upregulated in NPC biopsies (*p*-value < 0.0413) (Figure 4A), which further confirmed our analysis using the public datasets. To confirm that CACNA2D1 is specifically expressed in the NPC tumour cells, we clinically examined the expression level of CACNA2D1 using GeoMx digital spatial profiling (DSP). The clinical NPC patient FFPE slides were stained with DAPI, PanCK, and CD45 to locate the nucleus, normal and cancer epithelial cells, and immune cells, respectively. Inside the PanCK+ segments, CACNA2D1 showed a significantly higher expression level than the normal regions (*p*-value < 0.0182) (Figure 4B). After further assessing the expression of *CACNA2D1* at the single-cell level in NPC, single-cell molecular imaging (SMI) was used to profile clinically biopsied FFPE NPC samples in Guangzhou via the NanoString GeoMx^®^ DSP platform. In the images, NPC tumour regions show expression of CACNA2D1 at the transcript level. To quantify the results, the data show that *CACNA2D1* is significantly upregulated in NPC cells compared to normal controls (*p*-value < 0.0476) (Figure 4C). Immunohistochemistry (IHC) staining of CACNA2D1 was performed in clinical NPC patients to examine the expression of CACNA2D1 in matched normal and tumour regions scored by a pathologist. In line with the results, the tumour regions have a stronger staining intensity of CACNA2D1 than the adjacent normal regions, validated in all three NPC patient samples (Figure 4D). All these data pinpoint the specific expression of CACNA2D1 in NPC tumours.

### 2.4. CACNA2D1 Knockout Suppressed Cell Viability and Tumour Growth

**Figure 5:**
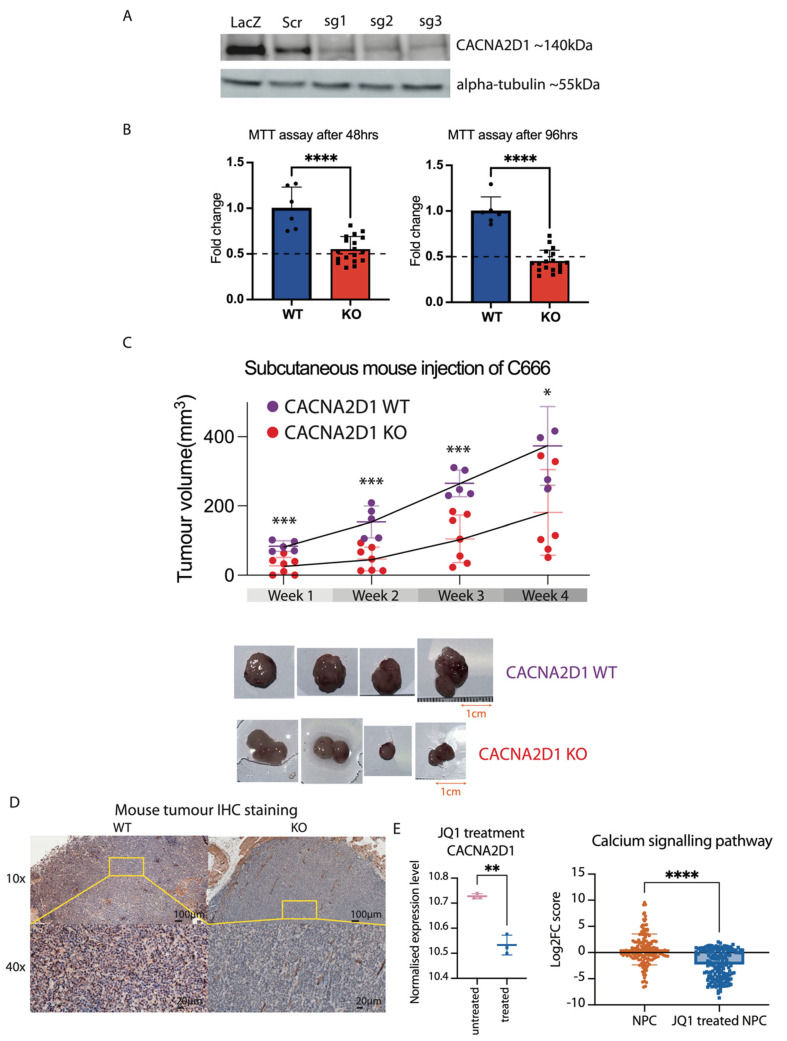
Functional characteristics of CACNA2D1 in vitro and in vivo experiments. (**A**) Establishment of knockout (KO) CACNA2D1 in the C666 cell line. Western blot was performed to evaluate the success of functional KO of CACNA2D1 in C666. The lacZ and scr control are wild-type (WT), and sg1, sg2, and sg3 are knockout (KO) clones. (**B**) MTT assay for WT and KO clones in C666. With an equal cell amount added to the 96-well plate, two independent MTT assays were carried out at 48 h and 96 h. The relative fold change was calculated based on the WT. An unpaired *t*-test was performed, and **** denotes a *p*-value < 0.0001. (**C**) Subcutaneous injection of WT and KO clones of C666 into nude mice. An equal number of cells for each clone was subcutaneously injected into the mouse. The growth of the tumour was measured continuously up to week 4. The length (L), width (W), and height (H) (mm) of the tumour were measured, and the total tumour volume was calculated based on L × W × H/2 (mm^3^). An unpaired *t*-test was performed for each week to test the statistical difference between WT and KO; * denotes *p*-value < 0.05, and *** denotes *p*-value < 0.001. (**D**) Two IHC staining results for nude mice at week 4. The mouse tumours were dissected in week 4, and IHC staining with CACNA2D1 antibodies was performed to confirm the results. (**E**) BRD inhibitor (JQ1) treatment suppressed the gene activity of *CACNA2D1* and the genes involved in the calcium signalling pathway. The RNA-seq data from 500 nM of JQ1 treatment in C666 for 72 h showed a significantly reduced *CACNA2D1* expression level. The hallmark of the KEGG pathway in the calcium signalling pathway was measured in NPC cell lines and the JQ1-treated C666 cell line. The score was measured for NPC cells versus normal control NPE cells. The JQ1-treated NPC was measured in JQ1-treated versus untreated cells in C666. The log2FC score plotted the fold change in genes in the calcium signalling pathway. The positive score indicates gene upregulation, and the negative score indicates gene downregulation. An unpaired *t*-test was performed for two box plots; ** adjusted *p*-value < 0.01, and **** adjusted *p*-value < 0.0001.

To assess the importance of CACNA2D1 in NPC tumour development, we selected a cell line, C666, with the strongest expression of CACNA2D1 to conduct the functional experiment. We established two wild-type (WT) and functional knockout (KO) cell clones via single-guided RNAs (sgRNAs) in the lentiviral CRISPR-Cas9 system. Western blot was used to examine the knockout efficiency (Figure 5A). As reported in many other cancers, the dysregulation of intracellular calcium ions in transmembrane transport plays a vital role in malignant transformation and cancer progression [52]. We performed an MTT assay to measure cellular metabolic activity in WT and KO cells. Indeed, a significant two-fold reduction in metabolic activity was exhibited in KO cells at both 48 and 96 h (*p*-value < 0.0001), implying that CACNA2D1 could increase tumour cell proliferation (Figure 5B). In addition to this, an assay was conducted to test the migration capability in 2D culture for WT and KO cells. With no significant difference on the initial day of the experiment, the wound healing assay showed that WT cells have a slightly stronger migration ability, with about a 1.2-fold change compared to KO cells after days 6 and 10 (Supplementary Figure S2C). We subsequently performed an in vivo experiment to further examine the functional role of CACNA2D1. With an equal amount of the cells administered subcutaneously to immunodeficient BALB/cAnN-nu (nude) mice via continuous monitoring for about four weeks, we found that the tumour size of WT mice grew significantly after week 1, by at least double, compared to the mice that did not express CACNA2D1. In the following two weeks, it was also shown that WT mice had far outgrown the KO mice, reaching statistical significance (*p*-value < 0.001) (Figure 5C). These mice were dissected at the end of week 4, and the mouse tumours were IHC stained with CACNA2D1 to confirm the KO efficiency, confirming that CACNA2D1 may provide tumour progression capability (Figure 5D).

Given that our data displayed enhancer infestation at CACNA2D1, we employed BRD inhibitor JQ1, which is known to inhibit the enrichment of BRD4 at enhancer sites [53]. After C666 cells were treated with 500 nM of JQ1 for 72 h, JQ1 impeded about 85% of the cancer cell proliferation (Supplementary Figure S2D). In particular, the expression level of *CACNA2D1* was significantly suppressed. Furthermore, JQ1 treatment demonstrated significant inhibition of calcium signalling-related genes, implying that the dysregulation in the calcium channel pathway can be treated with JQ1 (Figure 5E).

Taking the above results together, CACNA2D1 emerged as a primary candidate due to its specific upregulation in EBV-associated NPC (EBV+ NPC) cells. We also validated the elevated expression of CACNA2D1 in NPC tumours from patient samples. Additionally, CACNA2D1 knockout in NPC cells resulted in decreased cell viability and a reduced tumour volume in mice. Suppression of BRD4-bound enhancers resulted in decreased cell viability, suggesting that exploiting H3K27ac provides CACNA2D1 with a cancer cell growth advantage and therefore promotes cancer progression. Our findings underscore the potential of CACNA2D1 as a therapeutic target, warranting further investigation of its role in NPC progression and treatment.

## 3. Discussion

The global impact of using an AI-driven drug target screening platform in cancer research cannot be overstated. AI technologies, including machine learning, have transformed the drug discovery process by enhancing the efficiency and accuracy of identifying potential drug targets [22,54]. These platforms can analyse vast datasets, identify promising targets, and predict outcomes that would be challenging using traditional methods [20,24]. In this study, we leveraged an AI-driven drug target screening platform, PandaOmics, to identify CACNA2D1 as a potential oncogenic agent activated by enhancer infestation in Epstein–Barr virus-associated nasopharyngeal carcinoma (NPC). This innovative approach underscores the potential of AI in revolutionising cancer research and treatment strategies. The discovery of CACNA2D1’s activation through episomal EBV DNA tethering to the human genome, leading to enhancer infestation, not only provides new insights into NPC pathogenesis but also opens avenues for targeted therapeutic interventions.

The application of AI-driven platforms in drug discovery is particularly important in light of the current challenges faced in treating NPC. Although conventional therapies such as radiotherapy and chemotherapy have been effective in controlling the disease, they are not without limitations, including side effects, the development of resistance, and variable efficacy among patients [55,56,57]. Additionally, there is a lack of novel treatments for those with metastatic disease [58]. AI-driven drug discovery presents a promising solution by facilitating the identification of new therapeutic targets with the potential for reduced side effects with greater precision.

Our study employed a comprehensive approach that combined bulk RNA-seq, scRNA-seq, cross-species genome analyses, and functional validations. We demonstrated that CACNA2D1 was significantly upregulated in NPC across multiple datasets and experimental conditions, including bulk RNA-seq, scRNA-seq, and clinical biopsies. EBV episomes might tether to enhancer regions to drive CACNA2D1 expression, which was validated using a modified Hi-C technique, revealing significant genome rearrangements in EBV-infected NPC cells, particularly at the CACNA2D1 locus. Chromatin accessibility and histone modification profiling supported these findings, showing open chromatin and H3K27 acetylation at the CACNA2D1 promoter region. In vitro and in vivo functional assays confirmed that CACNA2D1 regulates tumour cell viability, migration, and tumour growth.

Enhancers are critical regulatory elements that govern gene expression, and their dysregulation can result in the activation of oncogenes and tumorigenesis [59,60]. The observation of enhancer infestation in CACNA2D1 and other candidate genes highlights the importance of enhancer dysfunction in cancer [61,62]. Through enhancer infestation, the EBV genome was implicated in the progression of NPC, which entailed interaction with inactive B compartments and the abnormal activation of enhancers [16]. In our study, the connection between EBV-interacting regions (EBVIRs) and enhancer infestation in CACNA2D1 suggests a mechanism by which EBV co-opts the cellular machinery of the host to facilitate NPC progression. Further knocking out the genomic region with EBV tethering on CACNA2D1 would be direct evidence to support that interactions of EBV episomes at the promoter region control the transcriptional upregulation of CACNA2D1.

CACNA2D1, a subunit of the voltage-gated calcium channel, plays a critical role in calcium transport, neuron functions, and the regulation of intraocular pressure [63,64,65]. The oncogenic role of CACNA2D1 has been demonstrated in various studies. Elevated expression of CACNA2D1 has been observed in head and neck squamous cell carcinoma tissue but not in normal tissue [66], and a high expression of CACNA2D1 has been associated with a poor prognosis in patients with gastric, breast, and epithelial ovarian cancers [51,67,68]. Furthermore, inhibiting CACNA2D1 has been shown to suppress cell proliferation and migration in endometrial cancer [69]. Similarly, CACNA2D1 depletion resulted in decreased colon cancer cell proliferation and migration, while also regulating fibroblast activities to influence the tumour microenvironment [70]. Calcium signalling is important in the control of cell proliferation. Spatiotemporal calcium signals activate transcription factors and several signalling pathways in a specific manner, including nuclear factor of activated T cells (NFAT) [71], cAMP response element-binding protein (CREB) [72], FOS, JUN, and MYC immediate early genes [73], Ras-extracellular-signal-regulated kinase (Ras-ERK) pathway [74], Oct/OAP, and Nuclear factor kappa B (NF-κB) [75,76]. The upregulation of CACNA2D1 likely increases calcium signalling in regulating these transcription factors and signalling pathways to promote tumour cell growth. In addition, CACNA2D1 has also been identified as a cancer stem cell marker for non-small-cell lung cancer and has been implicated in radiotherapy resistance through DNA damage repair regulation [37]. Our research demonstrated the activation of CACNA2D1 through enhancer infestation in NPC. Knocking out CACNA2D1 resulted in lower cell viability and reduced tumour formation in mice. The findings suggest that CACNA2D1 may serve as a promising therapeutic target for malignancies. Treatment with the BRD4 inhibitor—JQ1—could reduce *CACNA2D1* expression and suppress the calcium channel signalling pathway. The increase in the calcium channel signalling pathway may be linked to NPC tumour progression. JQ1 treatment suppresses the calcium channel signalling pathway and leads to cancer cell shrinkage.

Despite the promising findings, our study has limitations that warrant further investigation. The function of CACNA2D1 should be validated in organoid models and clinical trials to better confirm its therapeutic potential. As local recurrence and metastasis are the major causes of NPC-related death [77,78], the lack of a metastatic NPC model with which to investigate the role of CACNA2D1 is a limitation of this study. The most commonly used NPC cell lines (C666 and HK1) do not exhibit robust metastatic behaviour in vivo. Moreover, the orthotopic implantation of cells into the nasopharynx of mice is technically challenging due to its narrower size and hollow nature. Due to this, a liquid tumour cell suspension is difficult to access surgically or endoscopically, holding the injected tumour cell suspensions in the nasopharynx [79]. Humanised mouse models co-engrafted with NPC tumours with human immune system components or NPC organoids or spheroids in vitro 3D co-cultured with fibroblasts, immune cells, and endothelial cells could be utilised in the future for investigating the metastatic behaviour of CACNA2D1.

In conclusion, our study demonstrates the efficacy of an AI-driven drug target screening platform in identifying CACNA2D1 as an oncogene activated by enhancer infestation in EBV-associated NPC. This discovery not only advances our understanding of NPC pathogenesis but also offers a novel therapeutic target that could potentially improve treatment outcomes.

## 4. Materials and Methods

### 4.1. Bulk Transcriptomic Dataset Selection and Preprocessing

Publicly available microarray and RNA sequencing (RNA-seq) datasets for NPC and control samples were selected and retrieved from the Gene Expression Omnibus (GEO) database. This study includes GSE34573, Tay et al., GSE118719, GSE12452, GSE68799, GSE61218, GSE64634, GSE53819, and GSE13597. Only the human datasets with more than three NPC and three control samples obtained from the nasopharynx were selected. Since low tumour purity has been commonly observed for NPC sampling, all datasets were then screened for tumour purity using ESTIMATE [28]. The four datasets achieving acceptable purity (samples median ≥ 0.6) were then preprocessed (Supplementary Table S1). Microarray datasets were normalised using quantile normalisation, whereas RNA-seq datasets were normalised by upper-quantile normalisation. Gene IDs were mapped to the approved HGNC symbols [80].

### 4.2. Differential Expression Analysis and Meta-Analysis

For each dataset, differential gene expression analysis was performed by linear regression using the R package Limma (v3.54.2, 1 December 2022). Genes were considered differentially expressed with *p* < 0.05. The results of four case-control comparisons were then meta-analysed on PandaOmics, combining the log-fold change (logFC) and associated *p*-values using Stouffer’s method (Stouffer et al., 1949 [81]). The meta-analysis then combined multiple sources of evidence, such as genetic associations and pathway perturbation, to prioritise target genes for NPC treatment. After filtering for draggability, safety, confidence, and novelty levels [21], a list of the most promising target genes was selected and further assessed by downstream analyses.

### 4.3. Target Prioritisation by PandaOmics

PandaOmics is a cloud-based platform for target discovery that leverages artificial intelligence and deep learning to prioritise potential therapeutic targets. The process begins with transcriptomics data derived from NPC patient tissue samples. These experimental data are complemented by computational methods such as biological network analysis and AI-powered text mining, integrating evidence from scientific publications, clinical trials, and research funding sources to generate data-driven hypotheses. The system applies techniques like random walk algorithms on heterogeneous networks and non-negative matrix factorisation to generate a ranked list of candidate genes. A total of 23 independent models, covering omics, literature, financial interest, and expert validation, were used to evaluate the relevance of each gene to NPC. These models, scored from 0 to 1, were validated using a retrospective “Time Machine” approach and equally weighted in determining overall rankings. This ensemble modelling approach enhances both the robustness and reproducibility of target selection. We refined candidate lists using adjustable filters based on druggability, tissue specificity, gene family, and clinical development stage. Druggability was assessed in three key areas: small molecule accessibility, safety, and novelty. Accessibility was defined by whether the gene belongs to druggable families and whether known small molecules target its protein product, using data from Open Targets, ClinicalTrials.gov, and the Target Central Resource Database. Safety evaluations were based on gene essentiality and evidence from clinical trials. Novelty of each gene was quantified using a proprietary AI tool (available on https://pandaomics.com, accessed on 25 July 2022) that evaluated the number and content of the scientific literature that studied or discussed the gene in NPC. As a result, novel targets were those with limited biological characterisation or unclear roles in disease pathology. Genes from druggable protein families that were not classified as essential (per the Database of Essential Genes) were included to identify novel and therapeutically actionable targets. Comprehensive explanations of the scoring methods and filtering options can be found in the PandaOmics User Manual and the publication by Kamya et al. [21]. Additionally, Liu et al. provide a demonstration video illustrating the application of PandaOmics for target prioritisation in endometriosis [82]. The platform is offered as software-as-a-service at https://pandaomics.com (accessed on 25 July 2022).

### 4.4. Single-Cell RNA-seq (scRNA-seq) Data Analysis

To validate the expression of shortlisted genes at the single-cell level, a total of three publicly available scRNA-seq datasets were retrieved from the GEO database. The datasets were integrated and corrected for batch effects using the R package Seurat (v4) [83]. Sample annotations were retrieved from the corresponding original dataset. Cell types were annotated by the use of the top differentially expressed surface markers between clusters. Dimensionality reduction was performed using Uniform Manifold Approximation and Projection (UMAP). NPC cell-specific expression levels of each prioritised target gene were compared with other cell types and visualised by violin plots.

### 4.5. Hallmark Gene Set Enrichment Analysis

A total of 50 human hallmark processes with the associated gene list were retrieved from the MSigDB database using the R package msigdbr (v7.5.1) (Dolgalev, 2022 [84]). Geneset enrichment analysis (GSEA) was performed on these hallmark processes using the R package fgsea (v.1.24.0, 10 January 2023) (Korotkevich et al., 2019 [85]), for each of the four transcriptomics datasets. The resulting *p*-values and normalised enrichment score (NES) were combined using Stouffer’s method and arithmetic mean, respectively. All effect sizes and *p*-values were weighted equally across datasets.

### 4.6. Target Selection (CACNA2D1, NTRK2, PTGS2, ROBO1, DUSP10)

Based on multiple lines of evidence, targets were selected if they: (1) were ranked in the top-100 target list generated from the PandaOmics’ target identification engine; (2) were consistently differentially expressed with the same directional change (up- or down- regulation) across the four transcriptomics datasets; (3) were significantly dysregulated in both NPC and EBV+ NPC cell lines compared to controls; (4) showed NPC cell-specific expression in scRNA-seq data analysis; and (5) are safe and druggable based on the literature review.

### 4.7. Cell Lines

Two normal immortalised squamous epithelial cell lines derived from the nasopharynx were NP69 (RRID: CVCL_F755) and NP460-tert (NP460, RRID: CVCL_X205). The six NPC cell lines were C17 (RRID: CVCL_VT47), C666-1 (RRID: CVCL_M597), NPC43 (RRID: CVCL_UH64), NPC53 (RRID: CVCL_UH65), NPC38 (RRID: CVCL_UH63), and HK1 (RRID: CVCL_7084). All cells were cultured in a 37 °C incubator with 5% CO_2_. These cell lines were obtained from the Hong Kong AoE NPC Cell Line Repository at the University of Hong Kong. These cell lines were tested free from mycoplasma contamination and authenticated by STR profiling (Genetica and AoE NPC cell line repository).

### 4.8. Modified Hi-C to Identify EBV-Interacting Regions (EBVIRs)

A Dovetail^®^ Omni-C^®^ Library Preparation Kit (Cat# 21005) was used for the modified Hi-C experiment. The use of endonucleases (Dnase I) in the modified Hi-C experiment ensures the unbiased digestion of the chromatin compared to the traditional Hi-C, which uses restriction enzyme digestion. Following the recommended procedures in the manual, cells were freshly harvested and crosslinked. Chromatin was digested using the endonuclease enzyme. The chromatin was captured and re-ligated. Reverse crosslinking of the chromatin and fragmented chromatin was purified. Libraries were constructed using the Dovetail™ Library Module for Illumina (Cat# 25004, San Diego, CA, USA) and sequenced with 150 bp paired-end reads in the NovaSeq 6000 platform at the Centre for PanorOmic Sciences (CPOS) at the University of Hong Kong.

Alignment was performed in the BWA-MEM method to the human genome (hg19) and the EBV genome (NC_007605). With mapping quality MAPQ > 40, ligation junctions were identified using --walks-policy 5unique and --max-inter-align-gap 30 with Pairtools (v1.1.3, 1 October 2022). PCR duplicates were removed. Samples were performed in replicates to measure the reproducibility and data quality. Sequencing reads were normalised into 200 million paired reads by random shuffling. The contact frequency matrix was extracted using hic-straw with the Knight—Ruiz (KR) matrix balancing method at 1 kbp resolution. Regions of one side of the EBV genome to the other side of the human DNA genome were extracted. These interacting regions were ranked based on contact frequency to identify the high-confidence EBV-interacting regions (EBVIRs).

### 4.9. Cleavage Under Targets and Release Using Nuclease with Sequencing (CUT&RUN-seq)

The CUTANA^TM^ ChIC/CUT&RUN Kit (Cat# 14-1048) was used for the CUT&RUN-seq experiment. In brief, five hundred thousand cells were harvested and captured by concanavalin A-coated (ConA) magnetic beads. Cells were permeabilised with 5% digitonin, allowing the primary antibody to target histone modifications. The H3K27ac, H3K27me3, and mock IgG (Cat# 13-0042) antibodies were used. A 2.5 µL of CUTANA™ pAG-MNase (Cat# 15-1016) and 100 mM calcium chloride solution were used to fragment the chromatin. The enriched chromatin DNA was purified and prepared for sequencing libraries. The libraries were sequenced with 20 million 150 bp paired-end reads on the NovaSeq 6000 platform at the Centre for PanorOmic Sciences (CPOS) at the University of Hong Kong.

SNAP-CUTANA™ K-MetStat Panel (Cat# 19-1002) spike-in controls were used to measure the accuracy and noise-to-background ratio. The reproducibility of the sequencing data was monitored.

Trimmomatic (v0.39) was used to trim the adaptors. Bowtie2 (v2.5.0, 10 October 2022) was used to align reads to the human genome (hg19) and EBV genome (NC_007605). PCR duplicates were removed with Picard in GATK (v4.2.3.0). MACS2 was used to identify the peaks with matched control IgG with a q-value < 0.05. Sequencing signals were normalised in RPKM by deeptools (v3.5.6, 10 October 2022) in a 1 bp bin size.

### 4.10. The Assay for Transposase-Accessible Chromatin with Sequencing (ATAC-seq)

Tn5 Transposase (Illumina Cat# E7645S) was used for the tagmentation. Cells were lysed in cold lysis buffer (10 mM Tris-Cl pH 7.4, 10 mM NaCl, 3 mM MgCl_2_, 0.1% NP40). The transposition reaction took place at 37 °C for 30 min. The quantification PCR method was used to determine the number of PCR cycles required to avoid GC saturation of the signals. Sequencing was performed with 150 bp paired-end reads on the Illumina HiSeq X Ten platform.

The ENCODE ATAC-Seq pipeline (v1.0, 1 July 2022) was applied. Sequencing reads were adaptor-trimmed and aligned to the hg19 human genome. Peak calling and quality evaluation were evaluated. The featureCounts (v2.0.4, 1 July 2022) was used to count matrices for differential peak analysis in DESeq2 (v1.2.10, 1 July 2022). Absolute log2 fold change ≥ 2 and adjusted *p*-value < 0.01 were considered significant. Sequencing signals were normalised in RPKM by deeptools in a 1 bp bin size.

### 4.11. Validation of CACNA2D1 in NPC Cell Lines by Western Blotting

A standard Western blot experiment has been performed. In brief, cell lysates were collected and lysed using the RIPA buffer with proteinase and phosphatase inhibitors. Bradford protein assay has been conducted to normalise equal protein loading. Lysates were then heated up to unfold the secondary and tertiary protein structure. Sodium dodecyl-sulfate polyacrylamide gel electrophoresis (SDS-PAGE) has been carried out to separate the protein based on size. The gel was transferred to polyvinylidene difluoride (PVDF) membranes. Membranes were blocked by 5% BSA, and a ratio of 1:500 CACNA2D1 antibodies (MA3-921) (RRID: AB_2069904) was used for primary antibody detection. A ratio of 1:2000 dilution secondary antibody rabbit IgG antibody (HRP) (GeneTex Cat # GTX213110-01, RRID: AB_10618573, Irvine, CA, USA) was used to detect CACNA2D1. The α-tubulin protein was used as the loading control. The chemiluminescent protein detection method was used for protein detection.

### 4.12. Bulk RNA Sequencing (RNAseq) for Clinical Samples

NPC patients were recruited into the study at four public hospitals in Hong Kong, including Queen Mary Hospital, Queen Elizabeth Hospital, Princess Margaret Hospital, and Pamela Youde Nethersole Eastern Hospital. Informed consent was obtained for sample collection, and samples were collected according to protocols approved by the Institutional Review Board of the University of Hong Kong (UW 09-215).

RNA was extracted from fresh-frozen clinical NPC biopsies using the AllPrep DNA/RNA/miRNA Universal Kit (Qiagen, Hilden, Germany). The RNA samples were quantified using Qubit and Bioanalyzer (Agilent, Santa Clara, CA, USA). The library was prepared and sequenced on the Illumina NovaSeq platform by Novogene with 15 GB of raw data output per sample. The quality of RNASeq data was evaluated by FastQC (v0.11.8), and data were processed by our in-house pipeline using the reference human genome (hg38) by STAR (v2.7.5, 1 December 2022) and salmon (v1.3.0, 1 December 2022). Picard (v3.1.1, 1 December 2022) was used to evaluate the quality of the BAM files, and HTSeq was used to estimate the raw read counts. The read counts were input into the R package (v4.4.0, 1 December 2022) DESeq2 (v1.2.10, 1 December 2022) for downstream differential gene expression analysis.

### 4.13. Digital Spatial Profiling (DSP) for Spatial Transcriptome Analysis 

Clinically biopsied FFPE NPC samples were obtained from the Sun Yat-sen University Cancer Center in Guangzhou. Spatial human transcriptome profiling of the GZ TMA was performed on the NanoString GeoMx^®^ DSP platform targeting 18,000+ RNAs. The slides were first subjected to mIHC staining with fluorescent antibodies against PanCK and CD45, and mRNA probe incubation on the DSP platform. The RNA probes and antibodies coupled with photocleavable oligonucleotide barcodes were used to bind specifically to target mRNAs during the incubation process on the DSP platform on the basis of in situ hybridisation. Then, each segment was exposed to UV radiation, detaching the barcodes from the probes. The cleaved barcodes in units of segments were then collected into multiwell plates for next-generation sequencing on an Illumina sequencer. The counts of the oligonucleotide tags were finally used to calculate the abundance of the corresponding mRNAs. The quantile normalisation was performed for the transcriptome data for downstream differential gene expression analysis. Informed consent for sample collection was obtained, and samples were collected according to protocols approved by the Institutional Review Board of Sun Yat-Sen University Cancer Center (G2021-110-01).

### 4.14. NanoString CosMx^TM^ Single-Cell Molecular Imaging (SMI) for Single-Cell Spatial Analysis

Containing 1000+ RNA probes, together with custom in situ hybridisation probes targeting CACNA2D1, the SMI precisely locates the transcriptional expression data at a single-cell level. In situ hybridisation of RNA probes photochemically coupled with fluorescent barcodes in fields of view (FOVs), barcoded reads were being sequenced, and spatial images were generated with a subcellular resolution. The registered x, y, and z spatial positions were recognised based on the cleaved oligonucleotide tags. DAPI staining and antibodies were used to define the location of the nucleus and cell boundaries. Transcripts for each cell were obtained and processed using the AtoMx Spatial Information Platform (version 1.3.2). In this study, two NPC patients in FFPE slides obtained from Hong Kong Queen Elizabeth Hospital were used in the spatial analysis. The experiment was conducted by LifeStrands Genomics (Singapore).

### 4.15. Immunohistochemistry (IHC) Staining for Clinical Samples and Mouse Tumours

NPC patient FFPE tissue sections were used for the validation of the CACNA2D1 targets. Paraffin-embedded sections, 5 μm thick, were mounted on slides. Deparaffinised in xylene, tissue sections embedded in paraffin wax were dewaxed and rehydrated. To prevent nonspecific staining, endogenous enzymes were removed by immersing slides into 3% (*v*/*v*) H_2_O_2_ in ethanol with 0.06% (*v*/*v*) HCl for 30 min. Sections were washed with Tris-Buffered Saline (TBS) with 150 mM NaCl to rehydrate the slides. Heat antigen retrieval was achieved by immersing slides in 400 mL antigen retrieval buffer with 0.05 M Tris HCl, pH 9.5, preheated to 60 °C. Then, an 800 W microwave oven was used to irradiate for 5 min, followed by 1 min room temperature cool down. A volume of 200 μL 1% (*w*/*v*) BSA in 75% *v*/*v* TBS and 25% (*v*/*v*) normal serum was used to block the nonspecific protein interactions and secondary antibody host interactions. A ratio of 1:100 CACNA2D1 antibodies (MA3-921) (RRID: AB_2069904) was diluted in 1% (*w*/*v*) BSA in TBS for primary antibody detection. Sections were washed in TBS three times on a shaker. The biotinylated secondary antibody is applied for 30 min at room temperature for the detection. To form a “streptavidin–biotin” complex with the secondary antibody, an avidin–biotin complex (ABC) reagent was used. Sections were washed and hydrogen peroxide was applied in DAB in TBS for 20 min, forming a permanent dark brown precipitate. Sections were washed in distilled water for 5 min prior to adding Hematoxylin for 1 min and blued under running tap water for 5 min. The intensity of staining was scored as 0: negative, 1: weak, 2: moderate, and 3: strong. The quantity was scored as 100 times the positive percentage of the tumour cells in 10 increments. The intensity score was multiplied by the quantity score to generate the total score. All slides were scored by a senior pathologist blind to the grouping information.

### 4.16. Establishment of Functional Knockout CACNA2D1 by Genome Editing CRISPR-Cas9 System

In total, two wild-type controls and three knockouts targeting different exonic regions of the gene were designed. Oligo primer designs as follows, CACNA2D1 lacZ FP: 5′-CAC CGC TCT GGC TAA CGG TAC GCG TA-3′; CACNA2D1 lacZ RP: 5′-AAA CTA CGC GTA CCG TTA GCC AGA G C-3′; CACNA2D1 scr FP: 5′-CAC CGG TTC CGC GTT ACA TAA CTT A-3′; CACNA2D1 scr RP: 5′-AAA CTA AGT TAT GTA ACG CGG AAC C-3′; CACNA2D1 sg1 FP: 5′-CAC CGA TAA TAT CGA GCT AGG CCA G-3′; CACNA2D1 sg1 RP: 5′-AAA CCT GGC CTA GCT CGA TAT TAT C-3′; CACNA2D1 sg2 FP: 5′-CAC CGG AGC AAC AGA TCT AAA GCC C-3′; CACNA2D1 sg2 RP: 5′-AAA CGG GCT TTA GAT CTG TTG CTC C-3′; CACNA2D1 sg3 FP: 5′-CAC CGA TAT AGT AAA AAC TGT AGG T-3′; CACNA2D1 sg3 RP: 5′-AAA CAC CTA CAG TTT TTA CTA TAT C-3′. The oligos were annealed with a phosphate group at 37 °C for 30 min, followed by 70 °C for 10 min. Lenti-CRISPR-v2 plasmid has been used for cloning with BsmBI restriction enzyme cut sites. Annealed oligos were inserted and ligated into the plasmid at 16 °C overnight. The transformation took place with competent cells and ligation products in 1:10 with incubation in an LB Agar plate with 1:1000 ampicillin. Colonies were picked, and plasmid DNA was purified. The insertion of oligos into plasmid DNA was verified by Sanger sequencing. The 293T cells were used for lentivirus packaging, and control-GFP signals indicate the transfection efficiency > 80%. The infection took place with the virus, 500k culture cells, and Polybrene (10 mg/uL) in 400:800:2.4. After several days, the culture cells carrying plasmids were selected by puromycin (10 mg/uL). Western blotting was performed to indicate the success of the experiment. The protein expression levels of WT and KO cells were checked regularly to confirm the stability of the functional knockout.

### 4.17. MTT Assay

An equal number of 30 thousand cells was seeded into the 96-well plates in culture for treatment and control samples. Each set of experiments was performed in six replicates for each treatment and control sample. After cells were cultured for 48 h and 96 h, MTT reagent (5 mg/mL) mixed with distilled water was added into each well for the assay. The plate was incubated at 37 °C, 5% CO_2_ for 1 h. After washing with PBS, DMSO is added to the MTT assay to dissolve the formazan crystals. Formazan is determined spectrophotometrically at 570 nm.

### 4.18. Subcutaneous Injection of Mouse

The C666 cell line of four clones (wild-type and knockout) with replicates was subcutaneously injected with an equal number of four million cells into the immunodeficient BALB/cAnN-nu mice. The tumour size was measured by a calliper continuously within four weeks. The tumour volume was calculated by [length (L) × width (W) × height (H)]/2 in mm^3^. The experiment was granted ethical approval (CULATR 23-213), and the mouse was provided by the Centre for Comparative Medicine Research at the University of Hong Kong.

### 4.19. Ethical Statement

Informed consent was obtained for patient sample collection, and samples were collected according to the protocol approved by the Institutional Review Board of the University of Hong Kong (UW 09-215). All participants distributed and signed the consent form for participation. The animal work was granted ethical approval (CULATR 23-213) at the Centre for Comparative Medicine Research at the University of Hong Kong.

## Data Availability

The data presented in this study are available in GSE34573, 35394843 (Tay et al.), GSE118719, GSE12452, GSE68799, GSE61218, GSE64634, GSE53819, GSE13597. These data were derived from the following resources available in the public domain.

## Supplementary Materials

The following supporting information can be downloaded at: https://www.mdpi.com/article/10.3390/ijms26104697/s1.
