# Supplementary material for: AI-Driven Drug Target Screening Platform Identified Oncogene CACNA2D1 Activated by Enhancer Infestation in Epstein-Barr Virus-Associated Nasopharyngeal Carcinoma
Source: Int J Mol Sci. 2025 May 14;26(10):4697. doi: 10.3390/ijms26104697 (PMC12111453; doi:10.3390/ijms26104697)
Supplement: Supplementary file 1 [file ijms-26-04697-s001.zip › Supplementary Figures.pdf]

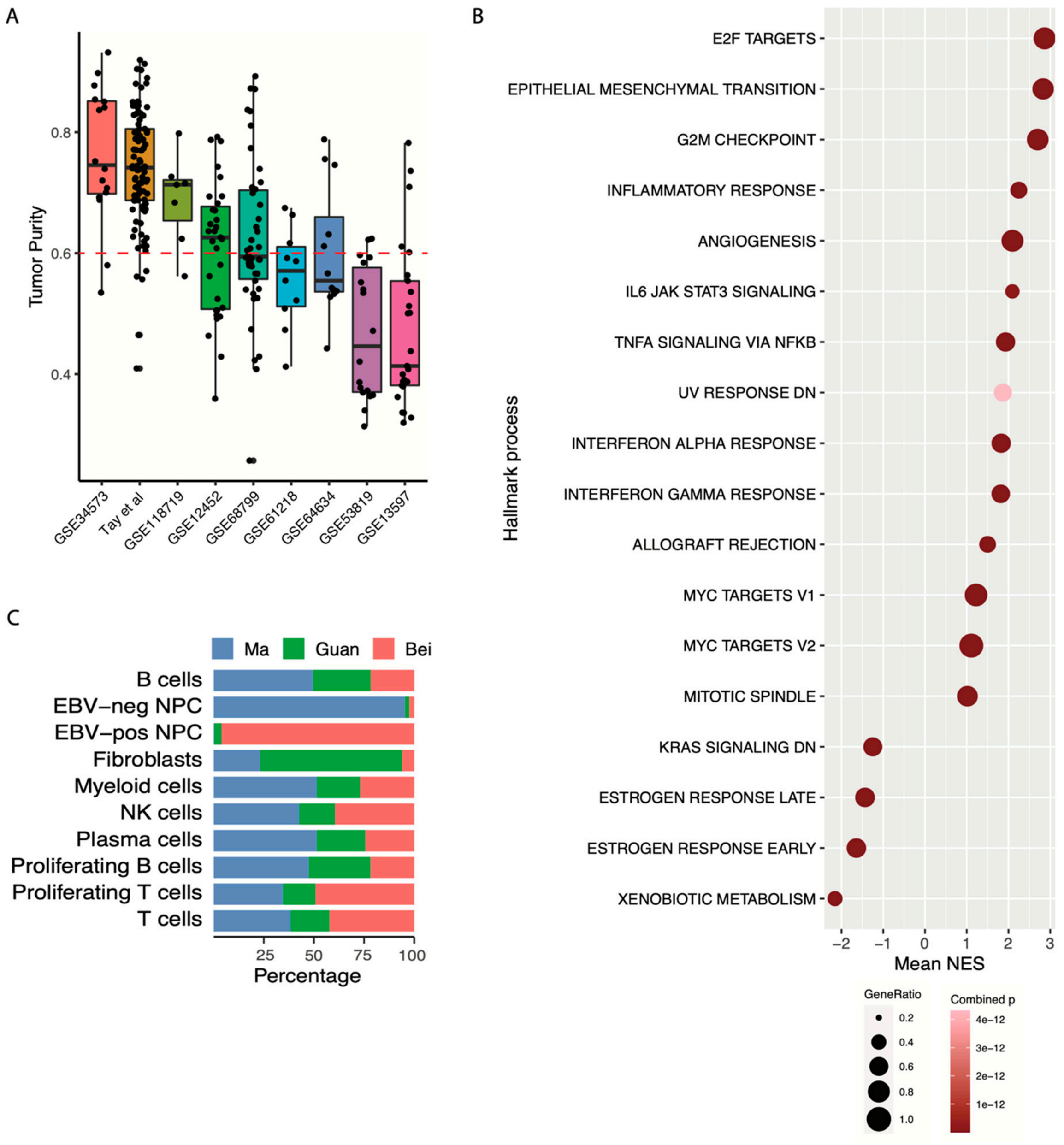

A
Tumor Purity
0.8
0.6
0.4
GSE34573
Tay et al
GSE118719
GSE12452
GSE68799
GSE61218
GSE64634
GSE53819
GSE13597
B
Hallmark process
E2F TARGETS
EPITHELIAL MESENCHYMAL TRANSITION
G2M CHECKPOINT
INFLAMMATORY RESPONSE
ANGIOGENESIS
IL6 JAK STAT3 SIGNALING
TNFA SIGNALING VIA NFKB
UV RESPONSE DN
INTERFERON ALPHA RESPONSE
INTERFERON GAMMA RESPONSE
ALLOGRAFT REJECTION
MYC TARGETS V1
MYC TARGETS V2
MITOTIC SPINDLE
KRAS SIGNALING DN
ESTROGEN RESPONSE LATE
ESTROGEN RESPONSE EARLY
XENOBIOTIC METABOLISM
−2 −1 0 1 2 3
Mean NES
GeneRatio
0.2
0.4
0.6
0.8
1.0
Combined p
4e−12
3e−12
2e−12
1e−12
C
Ma
Guan
Bei
B cells
EBV−neg NPC
EBV−pos NPC
Fibroblasts
Myeloid cells
NK cells
Plasma cells
Proliferating B cells
Proliferating T cells
T cells
25 50 75 100
Percentage

Supplementary Figure S1

A) Tumour purity for each NPC tumour sample in the nine publicly available bulk-RNAseq datasets, estimated using the ESTIMATE algorithm [1]. A threshold purity of 0.6 (red dashed line) was used to select the datasets for analyses based on their median values. A total of four datasets (GSE34573 [2], Tay et al. [3], GSE118719 [4], and GSE12452 [5]) were shown to have a median tumour purity of higher than 0.6 and were selected for analyses. **B)** Hallmark gene set processes that were dysregulated in the four bulk RNA-seq datasets in GSEA. The data shown demonstrate the combined results from the analysis performed on each of the four datasets. NES: normalised enrichment score. **C)** Distribution of cell types in the three publicly available scRNA-seq datasets. The three datasets exhibit different compositions for each cell type. Specifically, EBV- NPC cells were mainly obtained from Ma et al. [6], while EBV+ NPC cells were mainly from Bei et al [7].

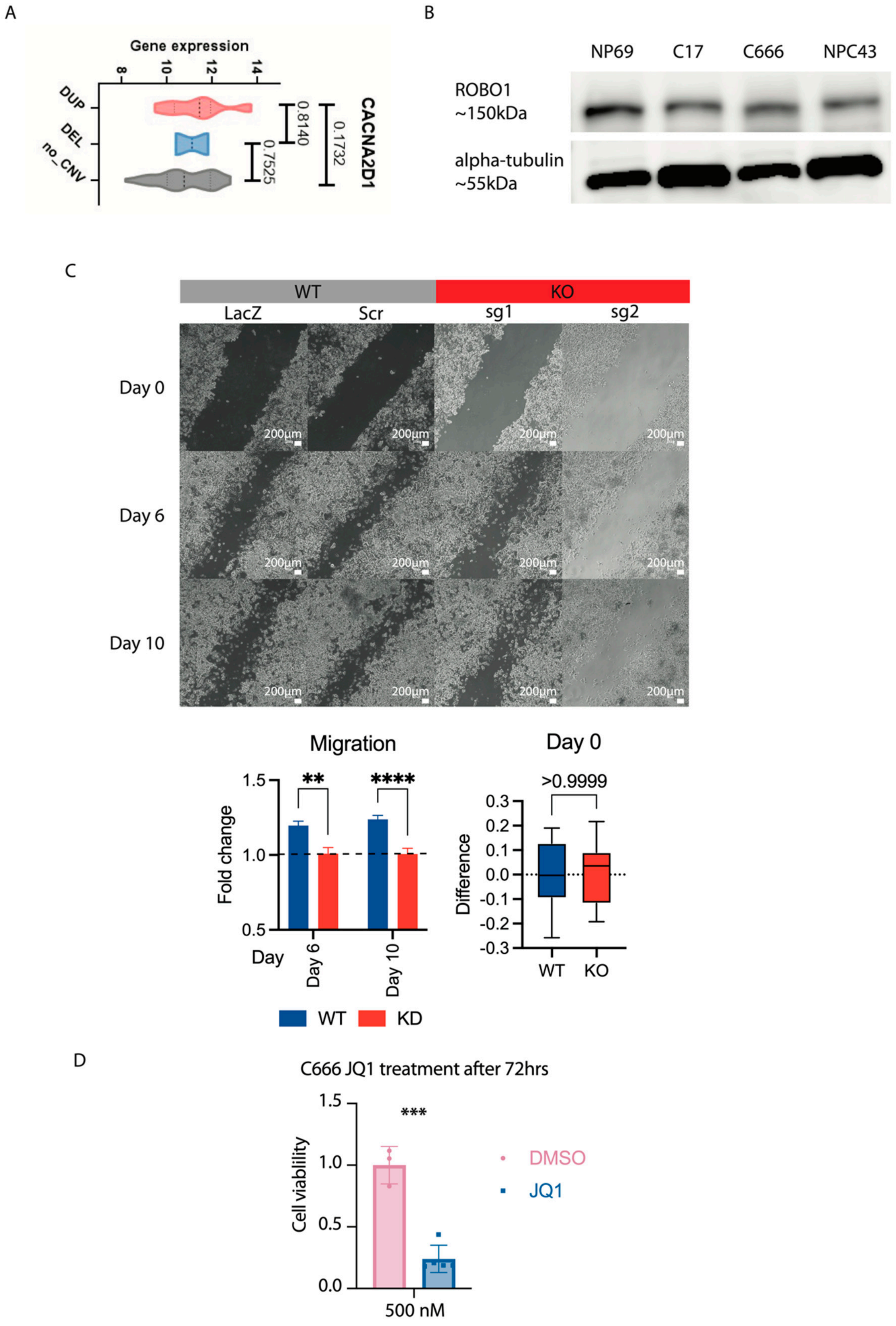

A
Gene expression
8
10
12
14
DUP
DEL
no_CNV
0.8140
0.7525
0.1732
CACNA2D1
B
NP69
C17
C666
NPC43
ROBO1
~150kDa
alpha-tubulin
~55kDa
C
WT
KO
LacZ
Scr
sg1
sg2
Day 0
Day 6
Day 10
200µm
Migration
Fold change
1.5
1.0
0.5
**
****
Day
Day 6
Day 10
WT
KD
Day 0
>0.9999
Difference
0.3
0.2
0.1
0.0
-0.1
-0.2
-0.3
WT
KO
D
C666 JQ1 treatment after 72hrs
Cell viability
1.5
1.0
0.5
0.0
***
500 nM
DMSO
JQ1

Supplementary Figure S2

A) Genomic alterations in CACNA2D1 and its expression level. Previously published WES data from 216 clinical patients with duplication (DUP), deletion (DEL), and no copy number variation (no_CNV) showed no statistically significant difference in CACNA2D1 expression levels [8]. A two-way ANOVA test was performed with Tukey correction, and p-values are indicated by each comparison. **B)** Protein expression levels for ROBO1. Western blot was carried out to evaluate the protein expression of ROBO1 at ~150 kDa in NP69 (control), C17, C666, and NPC43, with a housekeeping alpha-tubulin at ~55 kDa. The data show that both EBV+ NPC and normal epithelial cells expressed ROBO1. **C)** Migration assay in 2D culture for WT and KO clones in C666. The full confluency of the cells seeded in 12-well plates with three replicate measurements on day 0, day 6, and day 10. Scratches were created at equal distances for each well at day 0, and photos were taken under the microscope at the same 10x magnification. The average migration distance was measured using ImageJ software. A two-way ANOVA test was performed with Tukey correction; ** adjusted p-values of < 0.01 and **** adjusted p-values of < 0.001. **D)** MTT assay for 500 nM of JQ1 treatment in C666 after 72 hours. Treatments with 500 nM of DMSO control and JQ1 with replicates were carried out with an MTT assay. The cell viability was normalised to the DMSO control. An unpaired t-test was performed; *** denotes adjusted an p-values of < 0.001.

Reference

1. Yoshihara, K., et al., *Inferring tumour purity and stromal and immune cell admixture from expression data.* Nat Commun, 2013. **4**: p. 2612.
2. Hu, C., et al., *A global view of the oncogenic landscape in nasopharyngeal carcinoma: an integrated analysis at the genetic and expression levels.* PLoS One, 2012. **7**(7): p. e41055.
3. Tay, J.K., et al., *The microdissected gene expression landscape of nasopharyngeal cancer reveals vulnerabilities in FGF and noncanonical NF-kappaB signaling.* Sci Adv, 2022. **8**(14): p. eabh2445.
4. Lin, C., et al., *EBV-miR-BART8-3p induces epithelial-mesenchymal transition and promotes metastasis of nasopharyngeal carcinoma cells through activating NF-kappaB and Erk1/2 pathways.* J Exp Clin Cancer Res, 2018. **37**(1): p. 283.
5. Sengupta, S., et al., *Genome-wide expression profiling reveals EBV-associated inhibition of MHC class I expression in nasopharyngeal carcinoma.* Cancer Res, 2006. **66**(16): p. 7999-8006.
6. Chen, Y.P., et al., *Single-cell transcriptomics reveals regulators underlying immune cell diversity and immune subtypes associated with prognosis in nasopharyngeal carcinoma.* Cell Res, 2020. **30**(11): p. 1024-1042.
7. Liu, Y., et al., *Tumour heterogeneity and intercellular networks of nasopharyngeal carcinoma at single cell resolution.* Nat Commun, 2021. **12**(1): p. 741.

8. Dai, W., et al., *Clinical Outcome-Related Mutational Signatures Identified by Integrative Genomic Analysis in Nasopharyngeal Carcinoma.* Clin Cancer Res, 2020. **26**(24): p. 6494-6504.
